# Relational encoding of objects in working memory: Changes detection performance is better for violations in group relations

**DOI:** 10.1371/journal.pone.0203848

**Published:** 2018-09-11

**Authors:** Joel E. Bateman, William X. Q. Ngiam, Damian P. Birney

**Affiliations:** School of Psychology, University of Sydney, Sydney, New South Wales, Australia; Uppsala Universitet, SWEDEN

## Abstract

Research has indicated that working memory is based on forming relations between individual elements. In this study, we considered the congruency of object clusters during a change detection task. We demonstrate that changes which violate the relational encoding of a probe display (*single-object* changes where one object shifts independently from its corresponding group) are more easily detected than changes that maintain group structure (*cluster* changes where all objects in the group shift in location together)–despite cluster changes involving more objects moving overall. We explore this effect across interactions with direction of single-object movement (*distancing* from the cluster vs. *uniting* with the cluster) and trial order, demonstrating that naïve participants improve at a faster rate on single-object changes than cluster changes. It is concluded that storage in working memory functions by building relational bindings between objects and their place within the chunk, rather than by binding objects to their spatial location.

## Introduction

Change detection paradigms are employed to investigate visual short-term memory (STM) [1]. Using change detection, Jiang et al. [2] discovered participants tended to remember individual objects in relation to the object’s surroundings, even when the surroundings were task-irrelevant or when the target had been explicitly cued. This ‘relational grouping’ (encoding the configural relationships between objects into memory) has been shown to enhance recall [3, 4], suggesting that relational grouping is a necessary aspect of maintaining individual units of information (elements) in working memory (WM) [5–8]. In the current work, we examine the impact of relational grouping on change-detection performance by manipulating whether the change maintains the relational structure of the target group rather than changing the background stimuli.

The short-term memory system is responsible for maintaining temporary information in a highly accessible state over a short period of time (typically in the realm of seconds and minutes), whereas *working memory* [9] distinctly involves maintaining *and manipulating* information. This distinction is not often made in perceptual experiments [10] where visual WM is the preferred term. Visual WM research involves brief exposure times (less than one second) [11] and simple displays to assess immediate encoding performance while cognitive WM research typically allows participants to study elements [7]. Contemporary cognitive theories of WM see the maintenance and manipulation of information inherently intertwined [6, 7, 12], such that capacity limits in WM are simply limits on how information is integrated into chunks of information [7]. Although WM is often defined by the *manipulation* of information, cognitive WM theories posit chunk-formation involves relational integration processes. Given the importance of this integration process to cognitive WM theories, the current study focuses on this chunk-formation.

Cowan (7) suggests that chunks or the individual elements wherein are not directly related to the capacity of WM but are stored as a relation to some concept. For instance, recalling the sequence of letters “F-K-L” involves instantiating a relation to the concept of serial order. Similarly, Oberauer (6) proposes that information is maintained in WM by binding elements into a coordinated relational schema. For example, the recall elements F-K-L can be maintained through a schema of temporal order with F bound to temporal position *x*^1^ such that: F^1^, K^2^, L^3^. According to Oberauer, much of the strain on WM (and higher-order tasks) comes from having to flexibly bind and unbind elements in light of new, updated information. For instance, the *n*-back task [13] loads heavily on WM because it requires updating a running sequence of elements, with every new item presented requiring both binding of the new elements and unbinding of previously stored (but now unnecessary) elements. Halford et al. [8] hold a similar ‘binding’ view of WM but puts an emphasis on the contribution of processing limits to the ability to instantiate new relations. For Halford et al., the capacity of WM is limited by the maximum number of elements that must be simultaneously considered to comprehend the relation that connects them. For instance, comprehending a four-way interaction is considerably more complex than comprehending a three-way interaction, while comprehending a five-way interaction is virtually impossible [14]. Although these theories [6–8] each have some unique aspects, they share the view that WM capacity is based on *chunk-formation*. In these approaches, the ostensibly ‘un-manipulated’ maintenance of information is still subject to ‘processing-like’ limitations because the elements are stored via a common relation that must be instantiated.

Similar perspectives are found in the visual short-term memory literature on the importance of relational information. Vidal et al. [15] suggest that relational information is gleaned from the visual display and form a ‘structural gist’. Changing a feature of a non-target changes the ‘structural gist’ and impairs change detection. Similarly, Rensink [1] proposes that relational information between a set of objects is pooled into a nexus that contributes to higher-level decision-making (i.e., decisions about the group, rather than the object). The nexus is similar to the initial pooling of information in the structural gist process, though whereas the nexus exists as a separate source of information, the gist is bound with individual object information. The approaches are quite similar, though the nexus [1] suggests a more economical explanation, and accounts for the finding that it is easier to detect a change (among a group of non-changing objects) than it is to detect the absence of a change (among a group of changing objects) [16]. Despite this difference, both theories and the cognitive WM theories offer similar predictions on the importance of relational information.

Considerable work has been devoted to determining the nature of storage and processing limits, and how relational information changes this capacity [4, 17–20]. As noted however, the maintenance of elements through relations means that binding is an essential aspect to even basic storage-over-time tasks that have little higher-order processing. The impact of a relational binding theory of WM on even simple storage-over-time experiments is understated, because WM theories are typically concerned with explaining the link between WM and higher-order abilities such as reasoning or problem-solving [8, 21].

Consider Jiang et al. [2] who demonstrated that change detection for a single cued target worsened when unrelated background stimuli was altered between probe and test. Because the target’s surroundings were seemingly irrelevant to the single-target decision, the authors saw this an indication that relational information must be processed. One constraint on Oberauer’s [6] design is that elements must be bound into a relation to be mentally represented in WM. A singular object can be bound in a unary relation of space but there is no frame of reference with which to compare changes. By also binding the target object’s surroundings, altered relations between the object’s surroundings cue the observer that a change has occurred. Indeed, Jiang et al. [22] found that the poor performance associated with tampering with the surroundings could be attenuated by providing an invariant frame of reference (e.g., gridlines), providing an additional context for the target to be bound alongside.

**Fig 1:**
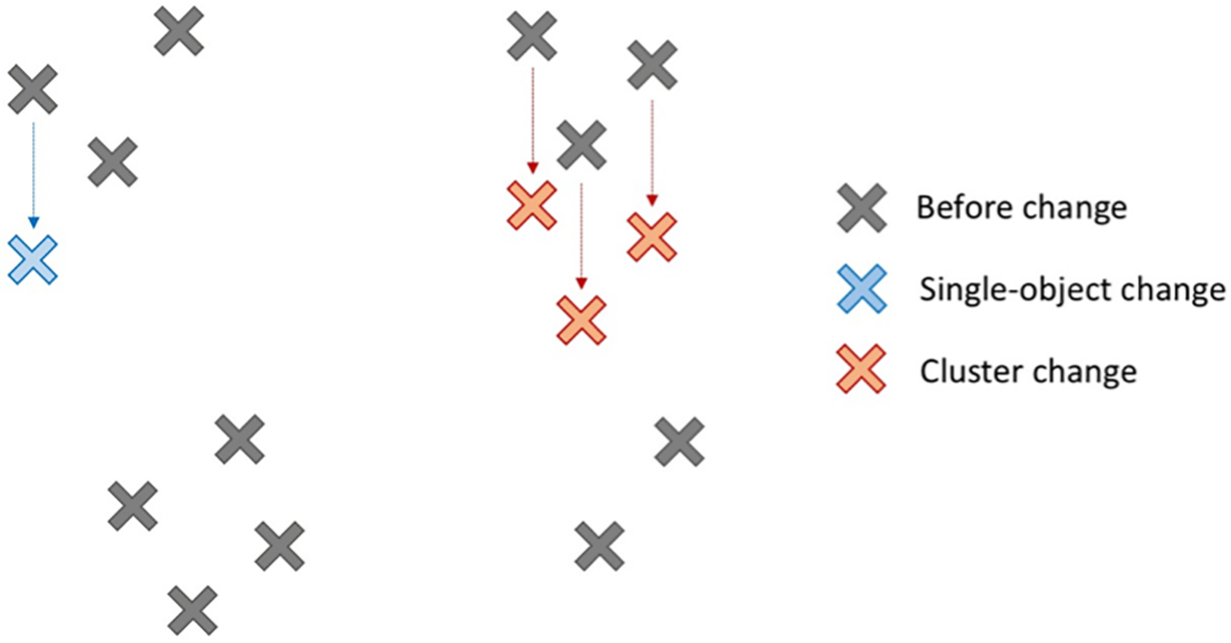
Example of relational encoding during the proposed change-detection task. Probe objects (grey crosses) are encoded as chunks of objects, due to proximity. The encoded relational information means a single-object change (indicated in blue) would be easier to detect than a cluster of objects changing (indicated in red), despite more objects overall moving in the cluster change.

Although Oberauer’s [6] cognitive-relational WM can account for these results, both Jiang et al. [2] and Jiang et al. [22] involved brief exposures (under 1 second) typically used when researching visual WM. Dent (23) employed longer exposure times (2 seconds) in the realm of cognitive WM, manipulating whether changes to a target object were coordinate-only (a shift in position that maintained relations between objects) or categorical (a shift in position that violated the categorical relationship, e.g., above-of became below-of). Despite both types of changes being identical in magnitude (in terms of change in visual angle), the categorical changes were detected at a higher rate than the coordinate changes. The displays were simple in nature (only four objects per display) and changes were always a single object moving. In the current study, we similarly employed longer display times but investigated change detection with multiple *clusters* of objects. This could help inform us of whether a group of objects is subject to similar limitations as coordinate changes. Consider Fig 1. If we assume individual objects are encoded and stored as a chunk, then we should see enhanced detection ability if the change occurs to a single object (the blue change in Fig 1), because it is inconsistent with the relations of the stored chunk. Alternatively, if a cluster changes (red change in Fig 1), the additional cues may provide better detection.

If WM is primarily based on relations, we would expect the single-object change to produce the greater detection rate. Unlike Dent [23], who focused on small set size displays and contrasted the position of two singular objects against one another, our displays involved large set sizes that clearly exceed the capacity of WM, but which could be grouped into manageable *clusters* of objects. We predict multi-object *cluster* changes, despite involving a change of a larger (surface) magnitude (i.e., more objects shift location), would be harder to detect than *single-object* changes as the spatial relation of the cluster is maintained. We designed the displays to encourage chunking of clusters: objects of the same cluster were the same shape (e.g., squares) and were closer in proximity to each other than to objects of other clusters [24, 25]. This encourages elaborated encoding: the high number of objects could be offset by grouping them into manageable chunks [26] that were clearly defined [7]. This encouraged chunking allowed more control over participants’ approach to the problem, mitigating the use of unconventional strategies like chunking with the borders which would contribute to error outside the core manipulation [7]. Because Dent’s [23] experiment was closest in nature to the current experiment, we also allowed participants multiple seconds to study the probe. Considering the increased set sizes of the displays relative to Dent, we varied probe durations at 3 and 5 seconds.

**Fig 2:**
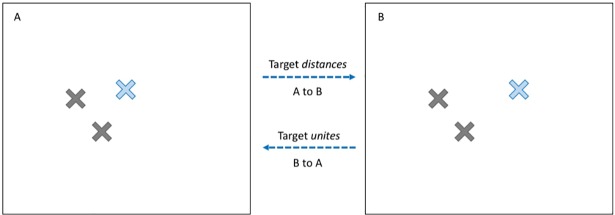
Example of the two types of single-object changes: *distancing* vs. *uniting*. If the display on the left (2A) and the display on the right (2B) is the test, then the target object has *distanced* itself from its cluster. Conversely, if 2B is the probe and 2A is the test, then the target object has *united* with its cluster.

Single-object changes can involve the target object shifted away or closer towards its cluster. Consider Fig 2. Changing whether 2A or 2B is the probe (and the other display is the test) varies whether the target is *distancing* or *uniting* relative to the cluster.

According to cognitive WM theories [6, 7], there is no particular reason to suspect that distancing or uniting should lead to different detection rates, because the relational information within the cluster is changing to a similar magnitude. However, if the cluster is initially easier to encode as a chunk (i.e., the probe is 2A), we would expect distancing changes to be easier to detect than uniting. Because the objects are more dispersed in 2B, the spatial relation might not be as easily encoded [27] and as such, the violation of the relation may be missed because the relation was more weakly encoded.

Typically, participants engage in practice items before a task. However, we were interested in comparing the trajectory of performance for naïve participants across the manipulations. If working memory is fundamentally relational [6], we would expect a large initial detriment for cluster change detection compared to single-object change detection, as cluster changes maintain the relation. However, over time, participants may learn to use more unconventional aspects (such as the screen border) which help specifically with cluster changes.

Previous studies have indicated that irrelevant objects in a display are still used to encode the position of target objects [22] and object position tends to be encoded categorically rather than using coordinates [23]. We extend this by considering change detection performance for multi-object clusters under exposure times that allow for elaborated encoding. We hypothesize that violations to the encoded relational structure (single-object changes) will be easier to detect than cluster changes, despite cluster changes involving a larger surface change to the display. This is in line with cognitive WM accounts [6–8], where independent elements (objects) are encoded as a relational structure (cluster) to form a chunk of connected information. We also extend this by comparing performance trajectories to determine which change type (single-object vs. cluster) is more intuitive to detect, hypothesizing that the difference between single-object and cluster changes is initially larger, but closes as participants learn to detect cluster changes. Finally, we hypothesize that distancing single-object changes will be easier to detect than uniting single-object changes, because the cluster’s relation to a more distant target object will be more weakly encoded than a close target object.

## Method

### Participants

Undergraduate students participated in the study as part of their psychology course and were asked at the end of the study whether they consented to contribute their data to further research purposes. This method of recruitment was approved by the Human Resources Ethics Committee at the University of Sydney. Only the data of those who consented are presented here. In total, 952 first-year psychology students (70.2% female) at the University of Sydney participated. The mean age was 19.42 (SD = 3.22) years. The large sample was the result of convenience. We acknowledge that this results in high power for the study, potentially exaggerating the results. As such, we reran the regression analyses five times using randomly selected subsets of the data (*n* = 200 each). The results of these analyses are reported in S1 Table. Overall, none of the main effects changed significance during any of these subsets. Interactions occasionally fell out of significance, though this was more due to increased variability in the confidence intervals than the size of the effect itself (odds ratio).

### Measure and procedure

Participants completed a change-detection task, programmed using *Inquisit Lab 5* [28] and administered via desktop computer. Participants were tested in groups of 15–25. Throughout the experiment, participants viewed a *probe* image consisting of various shapes for either 3 or 5 seconds. The *test* image was displayed following a 3 second inter-stimulus interval, and participants responded whether this test image was the *same* (using the ‘A’ key) or *different* (using the ‘L’ key) to the probe presented previously.

Items were designed such that 10–12 objects were arranged on an invisible 10 x 10 grid, centred on the screen. Each space on the grid was 2 x 2 cm and each object was 1 x 1 cm. Objects could not fall in the outer cells of the grid, but could appear on grid intersections. The objects were shapes of four kinds (circles, squares, triangles, crosses) and grouped into four clusters, one of each kind of shape. Objects of the same group were closer in proximity to each other than to objects of other groups. These design constraints were to facilitate grouping as a strategy to circumvent the otherwise large set size, allowing us to bias which groups were being formed by participants.

**Fig 3:**
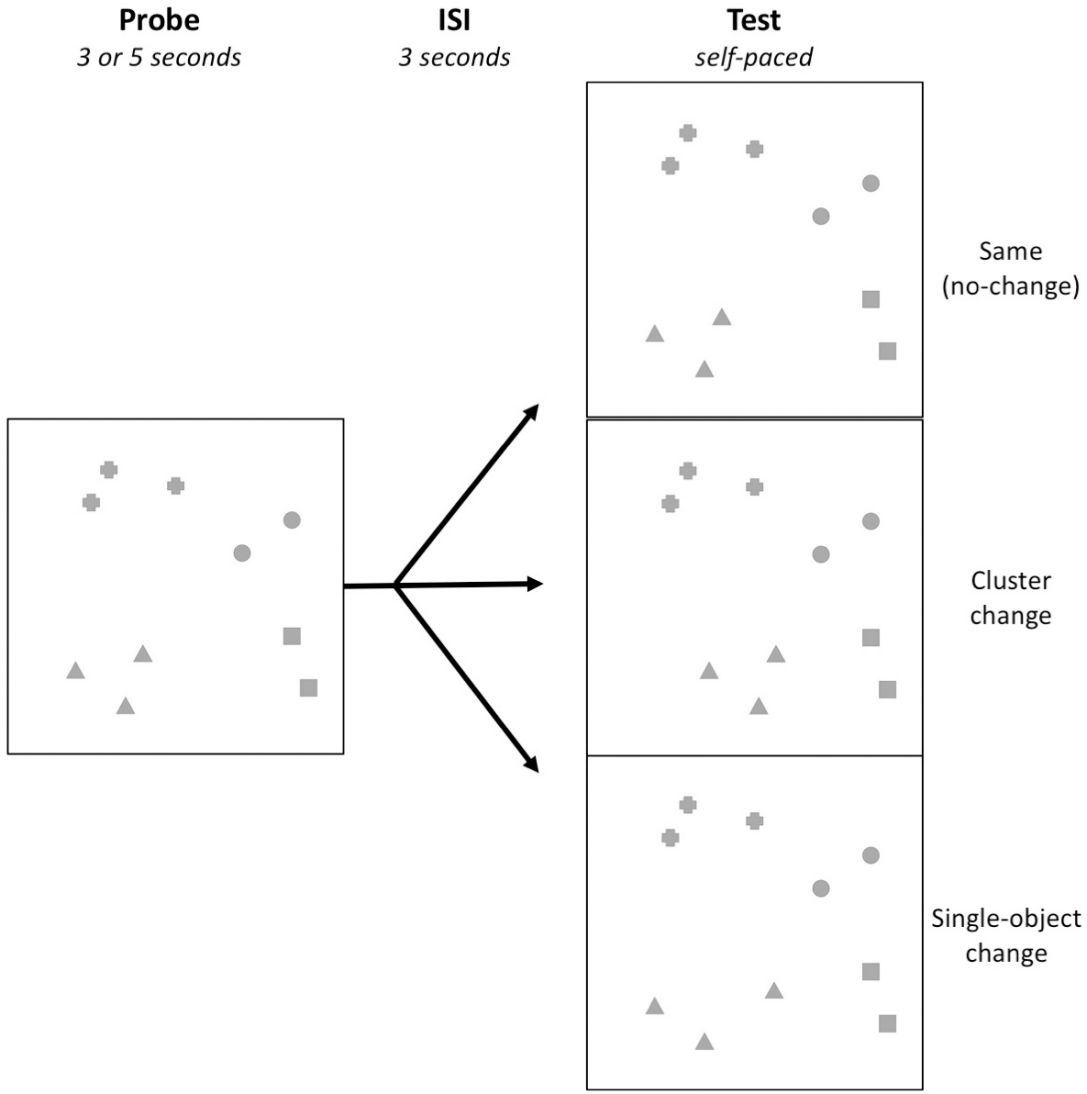
Example item demonstrating the three types of trials: same (no change), cluster change, and single-object change. In the example above, the target shape is triangle.

Participants first viewed task instructions which specified that the change would only concern location (objects moving, rather than changing identity) with demonstrations of both single-object and cluster changes. Twelve items were then administered. Half of the items were *no-change* trials and the other half were *change* trials. The change always involved one or more objects shifting location by 1.5 spaces (of the 10x10 grid; 2.5cm) in one of the eight cardinal or intercardinal directions. Pilot testing of different movement lengths indicated this was a sufficient degree of change to elicit responses above chance but below ceiling. Half of the *change* trials had single-object changes, where a single object changed location (independent of its cluster). The other half had cluster changes, where a clustered group of objects changed in the same direction together. Fig 3 demonstrates the change manipulation. Participants were randomly allocated to a direction condition, such that single-object changes for half the sample involved the object *distancing* from its cluster while for the other half, the object *united* towards its group.

## Results

**Fig 4:**
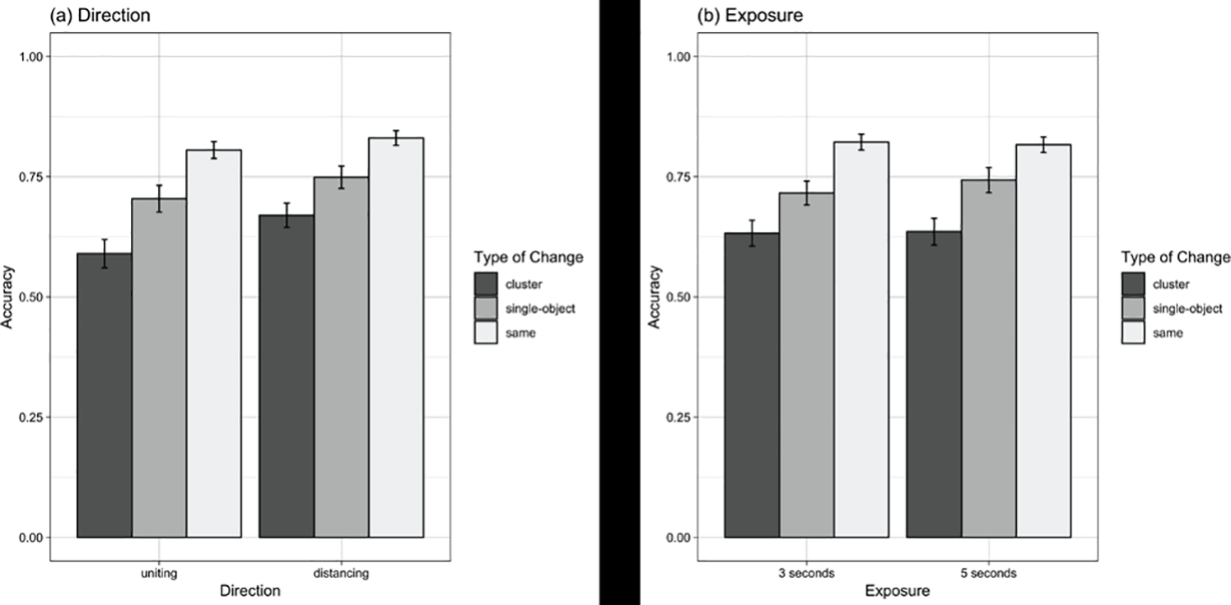
**Average (across all items) proportion correct for each type of change by (a) direction and (b) exposure.** Error bars represent 2 x standard errors.

All analyses were performed with R version 3.5.0 [29]. Plots were produced with the ‘sjPlot’ [30] and ‘ggplot2’ [31] packages. Hypotheses were tested by modelling item responses using a mixed-effects logistic regression approach as implemented in the ‘glmer’ procedure from ‘lme4’ (1.1.17) package [32]. In total, 952 participants provided 11,436 data points for analysis (excluding same items: 5,718 data points). The overall proportion of correct trials was .819 for same items, .635 for between-cluster changes, and .729 for within-cluster changes. Fig 4 demonstrates these proportions split across direction and exposure times. Collapsing over direction and exposure time, the difference between change types was statistically significant (χ^2^_2_ = 365.64, *p* < .001), such that accuracy was greater in same trials than single-object trials (OR = 2.65, se = 0.14, 95% CI [2.39–2.94], *p* < .001), and single-object accuracy was greater than cluster accuracy (OR = 1.56, se = 0.09, 95% CI [1.39–1.75], *p* < .001).

**Fig 5:**
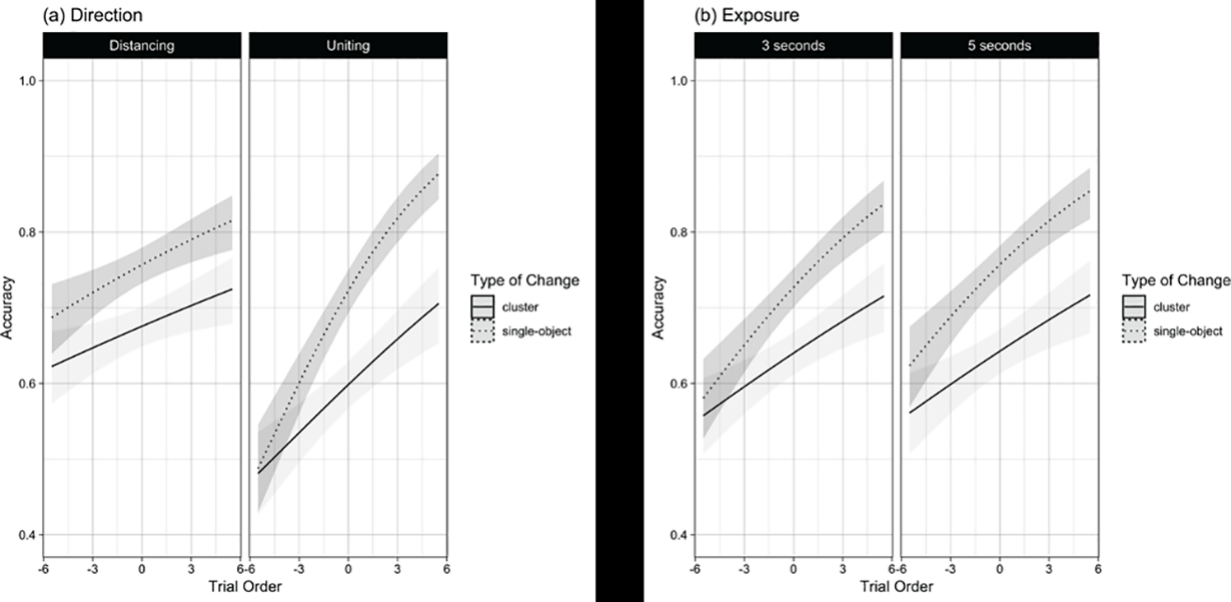
**Model plots of interactions (conditional on all other variables) for (a) direction, and (b) exposure.** Shaded areas represent 95% CI.

As the focus of our analyses is on differences between single-object changes and cluster changes, subsequent analyses excluded “same” items. All variables (i.e., change type (single-object, cluster), direction (distancing, uniting), exposure (3s, 5s), and trial order) and their interactions were regressed on accuracy (Fig 5). The regression coefficients are reported in Table 1. There was a significant main-effect for *direction* (OR = 0.77, se = 0.06 CI95% [0.67 – 0.89], p < .001), such that accuracy was higher for separating items than uniting items. There was no main-effect for *exposure* (OR = 1.13, se = 0.08, CI95% [0.98 – 1.30], p = 0.101) and *exposure* did not interact with any of the other variables (see Table 1). Trial order was a significant predictor of accuracy (OR = 1.07, se = 0.01, 95% CI [1.39–1.75], *p* < .001), with participants becoming more accurate in detecting change across the 12 items. This general improvement we refer to as the change-detection trajectory (CD-trajectory). Overall, the CD-trajectory was more pronounced for single-object changes than cluster changes (OR = 1.06, se = 0.02, CI95% [1.02 – 1.10], p = 0.001), although this effect was more pronounced for *direction* being uniting rather than distancing (OR = 1.08, se = 0.04, CI95% [1.01 – 1.16], p = 0.026). Simple-interaction analyses indicated that single-object CD-trajectories were significantly more pronounced than cluster CD-trajectories for uniting items (OR = 1.1, se = 0.03, CI95% [1.05 – 1.16], p = < .001), but not for distancing items (OR = 1.02, se = 0.02, CI95% [0.97 – 1.07], p = 0.460).

**Table 1:** Regression coefficients for all main effects and interactions.

	combined				uniting				distancing			
	*Odds Ratio*	*CI*	*std*. *Error*	*p*	*Odds Ratio*	*CI*	*std*. *Error*	*p*	*Odds Ratio*	*CI*	*std*. *Error*	*p*
**Fixed Parts**												
(Intercept)	**2.24**	**2.09 – 2.40**	**0.08**	**< .001**	1.96	1.77 – 2.18	0.1	**< .001**	2.55	2.32 – 2.81	0.12	**< .001**
direction (distancing vs uniting)	**0.77**	**0.67 – 0.89**	**0.06**	**< .001**								
exposure (3s vs 5s)	1.13	0.98 – 1.30	0.08	0.101	1.23	1.00 – 1.51	0.13	0.053	1.04	0.86 – 1.26	0.1	0.711
change (single-object vs cluster)	**1.62**	**1.44 – 1.82**	**0.1**	**< .001**	1.75	1.47 – 2.08	0.15	**< .001**	1.5	1.28 – 1.76	0.12	**< .001**
trial order (mean centered)	**1.1**	**1.08 – 1.12**	**0.01**	**< .001**	1.14	1.12 – 1.18	0.02	**< .001**	1.05	1.03 – 1.08	0.01	**< .001**
direction x exposure	1.18	0.89 – 1.56	0.17	0.255								
direction x change	1.17	0.92 – 1.48	0.14	0.2								
exposure x change	1.16	0.92 – 1.47	0.14	0.208	1.3	0.92 – 1.84	0.23	0.138	1.04	0.76 – 1.43	0.17	0.802
direction x trial order	**1.09**	**1.05 – 1.13**	**0.02**	**< .001**								
exposure x trial order	0.99	0.96 – 1.03	0.02	0.671	1	0.95 – 1.06	0.03	0.888	0.98	0.94 – 1.03	0.02	0.436
change x trial order	**1.06**	**1.02 – 1.10**	**0.02**	**0.001**	**1.1**	**1.05 – 1.16**	**0.03**	**< .001**	1.02	0.97 – 1.07	0.02	0.461
direction x exposure x change	1.25	0.78 – 2.00	0.3	0.357								
direction x exposure x trial order	1.02	0.95 – 1.10	0.04	0.54								
direction x change x trial order	**1.08**	**1.01 – 1.16**	**0.04**	**0.026**								
exposure x change x trial order	0.99	0.92 – 1.06	0.04	0.823	1.03	0.93 – 1.15	0.06	0.54	0.95	0.87 – 1.05	0.05	0.309
direction x exposure x change x trial order	1.09	0.94 – 1.25	0.08	0.256								
**Random Parts**												
τ_00, subject_	0.371				0.363				0.377			
N_subject_	952				426				527			
ICC_subject_	0.101				0.099				0.103			
Observations	5718				2556				3162			
Deviance	6221.116				2843.202				3377.964			

Note: all variables are mean-centered.

## Discussion

The current study contributes further evidence that WM stores and maintains information through relations. Single-object changes which violate the relation of grouped objects were more likely to be detected than cluster change where the relation is maintained. These results are consistent with Dent’s [23] findings on singular objects that categorical changes (changing relation) are easier to detect than coordinate changes (maintaining relation). Thus, although grouping efficiently maximises the amount of information that can be stored at any one time [4, 7], the present data indicates that this comes at a cost: visual changes where the relation between the shifted objects is maintained can be missed. This effect was demonstrated despite cluster changes involving changes of a larger veridical magnitude (more objects overall change location). This contributes to cognitive WM theories [6, 7] by suggesting that relational information is encoded and critical to the change detection process. As intended by the display arrangement, participants encoded surrounding objects as part of a structure, rather than situating the items at a particular coordinate. This can be extended to Jiang et al.’s [22] finding that changing task-irrelevant objects hinders performance not due to a general interference effect, but because this disrupts the structure of the display.

Using only a small number of trials but a large sample that was unfamiliar with the task allowed us to assess naïve approaches and how that changed across trials. Cluster changes were initially difficult to detect but participant performance improved rapidly over the 12 trials. Both uniting and distancing cluster changes had similar levels of performance by the end of the task. This suggests that participants were becoming aware of this type of change and potentially changed their approach to the task (possibly by using more higher-order encoding strategies, such as border anchors or labelling). Although performance for cluster changes was initially worse, single-object change detection performance improved at a faster pace. It must be cautioned that this interaction was qualified by a three-way interaction with the change type and trial order suggesting this improvement was faster for uniting trials than for distancing trials (Fig 5A). It appears that uniting single-object changes are initially more difficult to naïve participants than distancing single-object changes, indicating there may be effects of ease of initial encoding [27]. When objects are more disperse, it may be more difficult to form an accurate relation compared to when the objects form a regular structure. The violations with the uniting changes are then less likely to be noticed, not because they act as fundamentally different relations, but because they have never been encoded as the same chunk in the first place. Interestingly, participants improved in performance on *uniting* single-object changes faster than those detecting *distancing* single-object changes, indicating that these encoding effects are quickly overcome with task familiarity. That is, once participants were aware of the nature of the groups (all the same shape), they were able to encode the target as part of the cluster despite the increased distance. This theory could be confirmed by employing another condition where the target object starts out at distance from the group (like the uniting condition) but moves even further away. Thus, this condition would still be ‘distancing’ but the initial chunk to encode would also be distant. Alternatively, or in addition, the improvements may be a result of increasing understanding of task requirements and consolidation of more effective strategies, which may be prone to related individual differences that have not been explicitly investigated here.

We found no difference in overall change detection performance or learning trajectory when comparing display exposure times (3 vs. 5 seconds). This suggests that 3 seconds was sufficient time to consciously encode chunks despite having as many as three times the number of objects as Dent’s [23] displays. This is consistent with Rensink’s [4] finding that 12 items can be processed in approximately 1.5 seconds. The grouping cues of proximity and shape identity likely aided pooling of the objects [1]. If this is the case, then it is unlikely the extended probe duration equated to elaboration. Because both single-object and cluster performance improved over the course of the test, it is also possible that two levels of structure were formed simultaneously: one level encoding relations between items and one level encoding relations between clusters; with 3 seconds being sufficient to encode both levels. Hence, both single-object and cluster performance improved over time (albeit with single-object performance improving faster), because both levels of structure were being fine-tuned over the course of the task. If a lower exposure duration (e.g., 1 second) produced different trajectories (e.g., single-object performance improves, but cluster performance does not), it would confirm that two levels of structure are present.

It should be cautioned that the learning trajectories presented here are based on naïve participants and limited to 12 trials. Because performance did not reach asymptote in either single-object or cluster conditions, it is possible that performance trajectories could continue or change with additional trials. What we have demonstrated is that the learning trajectories of the two types of changes start out similar and quickly grow dissimilar (standard errors between the conditions generally lose their overlap by the third trial). Although we cannot say if this trend continues past 12 trials, it does indicate that initial learning of the task widens the gap between detection rates of single-object and cluster changes, supporting the conclusion that detection of changes which violate relational structures is learned more intuitively than changes which maintain relational structure.

It is clear that structure and relation are critical to memory [6–8] and form the cornerstone of higher-order intelligence [21, 33, 34]. Oberauer [6] suggests that representations are only maintained within immediately accessible memory by the binding of an individual element to a context within a relational schema. As a result, actively maintaining elements is dependent on relational information. While single-object change detection was relatively better than cluster change detection, we cannot conclude that memory is entirely dependent on this information. Further, because our experiment was based on changes in spatial position, we cannot necessarily generalize these findings to other visual properties or verbal information. Nonetheless, the present results, together with a cognitive approach to WM (extending on more perceptual accounts of visual WM), produces interesting implications for our understanding of the process and constraints involved in grouping spatial information. The current results indicate that grouping information is an effective way to bypass capacity limits, but it comes at a cost: changes that maintain the relational structure of the display are more likely to go undetected. Multi-object groups can shift unbeknownst to participants if their spatial relation is maintained. Like a guard falling out of line with the marching drill, a single object changing independently of its group is conspicuous. It appears that maintaining information in WM is dependent on the relations that connect that information.

## Supporting information

S1 Table Regression analyses on random subsets of data (*n* = 200 each). (DOCX)
