# Correction: Relational encoding of objects in working memory: Changes detection performance is better for violations in group relations

**DOI:** 10.1371/journal.pone.0209207

**Published:** 2018-12-10

**Authors:** Joel E. Bateman, William X. Q. Ngiam, Damian P. Birney

In the title of this article, the word “Changes” should be “Change.” The correct title is: Relational encoding of objects in working memory: Change detection performance is better for violations in group relations. The correct citation is: Bateman JE, Ngiam WXQ, Birney DP (2018) Relational encoding of objects in working memory: Change detection performance is better for violations in group relations. PLoS ONE 13(9): e0203848. https://doi.org/10.1371/journal.pone.0203848.
