# Supplementary material for: Relational encoding of objects in working memory: Changes detection performance is better for violations in group relations
Source: PLoS One. 2018 Sep 11;13(9):e0203848. doi: 10.1371/journal.pone.0203848 (PMC6133376; doi:10.1371/journal.pone.0203848)
Supplement: S1 Table — (DOCX) [file pone.0203848.s001.docx]

Supporting information

**S1 Table. Regression analyses on random subsets of data (*n*=200 each)**
